# IL-26 Kick-Starts Rheumatoid Arthritis

**DOI:** 10.1371/journal.pbio.1001398

**Published:** 2012-09-25

**Authors:** Caitlin Sedwick

**Affiliations:** Freelance Science Writer, San Diego, California, United States of America

**Figure:**
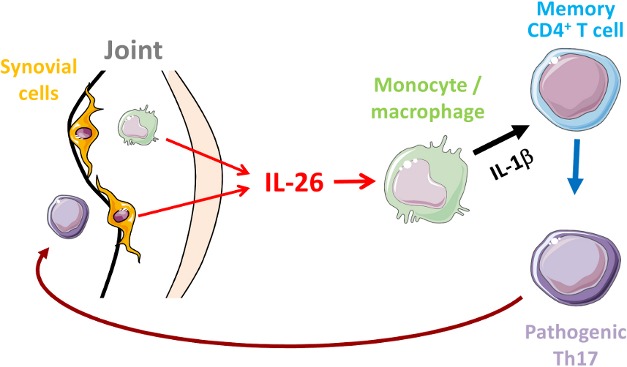
Schematic representation of the role of IL-26 on the generation of pathogenic Th17 cells in rheumatoid arthritis.

Rheumatoid arthritis (RA) is an autoimmune disease that afflicts millions of people worldwide, wherein cells of the immune system attack tissues that seem otherwise healthy, causing pain and tissue damage. Usually the immune cells attack the joints, although other tissues may come under attack as well. What instigates this assault, and what governs its spread to previously unaffected tissues, is currently unknown. In this issue of *PLOS Biology*, Murielle Corvaisier, Pascale Jeannin, and colleagues describe a new inflammatory cascade that contributes to early stages of RA disease development.

It's well known that inflammation is at the heart of RA disease. This is true even though under normal conditions, inflammation is helpful to the body. It protects the body against pathogens and other forms of tissue damage by recruiting immune cells to kill pathogens and clear away injured or dying body cells. As clean-up proceeds, the stimuli that support inflammation disappear, and the inflammatory response gradually fades away. But in RA, where immune cells are somehow induced to attack normal body tissues, the inflammatory stimulus is permanently present, so the inflammation never resolves. As a result, affected tissues become jam packed with immune cells all bent on attacking their surroundings. In joints, this results in swelling, stiffness—and, when damage has advanced far enough—inability to bend the joint.

Multiple inflammatory processes and immune cell types appear to be simultaneously at work in RA. In particular, myeloid cells (which include every kind of immune cell that is neither a T nor B lymphocyte) are thought to orchestrate much of the disease process. But another immune cell type recently shown to contribute to RA is the so-called Th17 cell. This is a kind of T lymphocyte that produces a secreted protein, the cytokine IL-17, which in turn strongly stimulates pro-inflammatory activities in other immune cell types and in fibroblasts present in RA joints.

Because Th17 cells are important to progression of RA disease, Corvaisier and colleagues became curious about why Th17 are cells present in RA joints in the first place. Studies of a different autoimmune disorder (Crohn's disease) by other research groups had suggested that Th17 cell infiltration into affected tissues was linked to another cytokine, IL-26. Corvaisier et al. therefore set out to see whether IL-26 might contribute to RA disease development.

Because the gene encoding IL-26 is absent from rodents, the researchers conducted their studies in humans. They quickly found evidence supporting the idea that IL-26 contributes to RA: compared to serum and synovial fluid from healthy individuals, serum from RA patients and synovial fluid from RA-affected joints both exhibit elevated levels of IL-26. They also found that IL-26 is produced by fibroblast-like cells called synoviocytes. Although synoviocytes are present in all joints, only synoviocytes from RA patients can produce IL-26.

What are the consequences of IL-26 production in RA joints? To find out, the authors examined what happens to various types of immune cells upon exposure to IL-26 in vitro. These experiments showed that myeloid cells, and in particular a subset of myeloid cells called monocytes, ramp up their production of pro-inflammatory cytokines and chemokines after exposure to purified IL-26. Crucially, one of the cytokines produced by IL-26-treated monocytes causes certain T lymphocytes, called memory T cells, to convert into Th17 cells. Consistent with this observation, synovial fluid taken from RA joints can also help monocytes drive T lymphocytes' conversion into Th17 cells. But this effect is lost when IL-26-blocking antibodies are added to RA synovial fluid.

Corvaisier et al. conclude that synoviocytes secreting IL-26 cause monocytes to secrete factors that promote the accumulation of Th17 cells. And in an interesting twist, the authors also found that IL-17 promotes IL-26 secretion by synoviocytes, suggesting these factors can operate in a positive feedback loop. Such amplification could explain how this early inflammatory process is sustained and eventually develops into a damaging chronic disease. It remains unclear why synoviocytes begin producing IL-26 in the first place, but that is something that will have to be addressed in future work. Nonetheless, the authors suggest, it would be worthwhile to investigate whether interfering with IL-26 production—perhaps by introducing IL-26-blocking antibodies to affected joints early in the disease—might short-circuit RA disease progression.

Finally, these findings have implications beyond RA. Other autoimmune inflammatory disorders, including but not limited to Crohn's disease, might operate on similar principles as RA. Further work is needed to explore this possibility.

**Corvaisier M, Delneste Y, Jeanvoine H, Preisser L, Blanchard S, et al. (2012) IL-26 Is Over-Expressed in Rheumatoid Arthritis and Induces Proinflammatory Cytokine Production and Th17 Cell Generation. doi:10.1371/journal.pbio.1001395**

